# *Pde3a* and *Pde3b* regulation of murine pulmonary artery smooth muscle cell growth and metabolism

**DOI:** 10.14814/phy2.70089

**Published:** 2024-10-22

**Authors:** Paulina N. Krause, Gabrielle McGeorge, Jennifer L. McPeek, Sidra Khalid, Leif D. Nelin, Yusen Liu, Bernadette Chen

**Affiliations:** ^1^ Center for Perinatal Research Abigail Wexner Research Institute at Nationwide Children's Hospital Columbus Ohio USA; ^2^ Department of Pediatrics The Ohio State University College of Medicine Columbus Ohio USA

**Keywords:** AMPK, CREB, metabolic, PDK, PGC‐1α, PPARγ, pulmonary hypertension, vascular remodeling

## Abstract

A role for metabolically active adipose tissue in pulmonary hypertension (PH) pathogenesis is emerging. Alterations in cellular metabolism in metabolic syndrome are triggers of PH‐related vascular dysfunction. Metabolic reprogramming in proliferative pulmonary vascular cells causes a metabolic switch from oxidative phosphorylation to glycolysis. PDE3A and PDE3B subtypes in the regulation of metabolism in pulmonary artery smooth muscle cells (PASMC) are poorly understood. We previously found that PDE3A modulates the cellular energy sensor, AMPK, in human PASMC. We demonstrate that global *Pde3a* knockout mice have right ventricular (RV) hypertrophy, elevated RV systolic pressures, and metabolic dysfunction with elevated serum free fatty acids (FFA). Therefore, we sought to delineate *Pde3a/Pde3b* regulation of metabolic pathways in PASMC. We found that PASMC *Pde3a* deficiency, and to a lesser extent *Pde3b* deficiency, downregulates AMPK, CREB and PPARγ, and upregulates pyruvate kinase dehydrogenase expression, suggesting decreased oxidative phosphorylation. Interestingly, siRNA *Pde3a* knockdown in adipocytes led to elevated FFA secretion. Furthermore, PASMC exposed to siPDE3A‐transfected adipocyte media led to decreased α‐SMA, AMPK and CREB phosphorylation, and greater viable cell numbers compared to controls under the same conditions. These data demonstrate that deficiencies of *Pde3a* and *Pde3b* alter pathways that affect cell growth and metabolism in PASMC.

## 1 INTRODUCTION

Pulmonary hypertension (PH) is a heterogenous, multi‐factorial disease characterized by increased pulmonary vascular resistance with progression to increased right ventricular afterload, right heart failure and death, if left untreated. The hallmark features of PH include vascular cellular proliferation and remodeling that can occur at all layers of the vessel wall (Humbert, Sitbon, & Simonneau, 2004). Invasion and abnormal proliferation of the endothelial cell layer and smooth muscle cell (SMC) layer of the pulmonary vessel wall can be seen with extension of smooth muscle into smaller, normally non‐muscularized pulmonary arteries, leading to decreased vessel lumen diameter and increased resistance to blood flow (Humbert, Morrell, et al., 2004; Pietra et al., 2004). In addition, the pulmonary vascular cells become resistant to apoptosis and develop a glycolytic shift from oxidative phosphorylation towards less efficient metabolic pathways, thus supporting the idea that metabolic disturbances contribute to vascular remodeling (Culley & Chan, 2018; Lechartier et al., 2022; Paulin & Michelakis, 2014; Ryan et al., 2015). These described processes lead to the progressive nature of a devastating disease with limited therapies that target vasoconstriction rather than prevent and/or reverse the pathologic vascular remodeling associated with PH.

Cyclic nucleotide phosphodiesterases (PDE) hydrolyze cAMP and/or cGMP, which are critical second messengers involved in many cellular processes via regulation of signal transduction. Both cyclic nucleotides play a role in the vasodilatory and proliferative responses within the pulmonary vasculature, which is important in PH pathophysiology (Humbert, Sitbon, & Simonneau, 2004). PDE3, of which there are two known subtypes – PDE3A and PDE3B, is one of the predominant PDE families that catalyze cAMP and cGMP degradation in the pulmonary vasculature (Dillard et al., 2020; Murray et al., 2002; Thompson et al., 2007). We and others have shown that PDE3 can be regulated by the nitric oxide – cGMP signaling pathway in the pulmonary vasculature (Busch et al., 2010; Chen, Lakshminrusimha, et al., 2009; Dillard et al., 2020). Both PDE3A and PDE3B exhibit varying patterns of expression and are postulated to serve different cell‐specific functions. PDE3A is generally considered as the principal subtype in the regulation of cardiac and vascular smooth muscle contractility (Begum et al., 2011; Chung et al., 2015; Sun et al., 2007), whereas PDE3B is important in the regulation of energy metabolism (Chung et al., 2017; Degerman et al., 1997). Both PDE3A and PDE3B subtypes have been shown to regulate AMP‐activated protein kinase (AMPK), an ubiquitously expressed serine/threonine protein kinase that acts as a sensor of cellular energy status and is activated in response to metabolic stresses (Chung et al., 2017; Dillard et al., 2020; Hardie, 2011). AMPK is increasingly being identified as an important contributor to the pathophysiology seen in PH (Afolayan et al., 2016; Lai et al., 2016; Omura et al., 2016; Teng et al., 2013). Our data demonstrate that deficiencies of PDE3 subtypes alter pathways that affect growth and metabolism in PASMC. Additionally, our data show that *Pde3a*‐deficient mice have right ventricular (RV) hypertrophy and associated high RV systolic pressures, as well as elevated serum free fatty acids (FFA), a contributing factor to metabolic dysfunction.

Although the PDE3 inhibitor milrinone is a commonly used medication in the neonatal PH population for heart failure aiding in inotropy and pulmonary vasodilation (Bassler et al., 2010; Cookson et al., 2022; McNamara et al., 2013; Opie, 1986), there are no randomized clinical trials to establish its therapeutic efficacy in PH. Additionally, milrinone inhibits both PDE3A and PDE3B. The present study seeks to differentiate the roles of PDE3A and PDE3B in the regulation of metabolic pathways and the proliferation of pulmonary artery (PA) smooth muscle cells (PASMC). The results presented here demonstrate that PDE3A and PDE3B function to regulate metabolic pathways and cell growth in PASMC.

## 2 MATERIALS AND METHODS

### 2.1 Mice

*Pde3a* ^−/−^ male and *Pde3a* ^−/+^ female mice (Beca et al., 2013; Masciarelli et al., 2004) on a C57BL/6J background and male and female *Pde3b* ^−/−^ mice on a C57BL/6NTac background (Choi et al., 2006; Guirguis et al., 2013) were originally received from the laboratory of the late Dr. Vincent Manganiello at the National Institutes of Health. To establish mouse strains with identical genetic background, *Pde3a* ^−/−^ and *Pde3b* ^−/−^ were backcrossed nine generations to C57BL/6J (Jackson Lab, Bar Harbor, ME) to produce *Pde3a* ^−/−^ (3A‐KO) and *Pde3b* ^−/−^ (3B‐KO) on a pure C57BL/6J background. Because female *Pde3a* ^−/−^ are sterile (Masciarelli et al., 2004), *Pde3a* ^−/−^ mice were generated by crossbreeding between male *Pde3a* ^−/−^ and female *Pde3a* ^*+/−*^ mice or between male and female *Pde3a* ^*+/−*^ mice. *Pde3b* ^−/−^ mice are not sterile and were generated by continued breeding of male and female *Pde3b* ^−/−^ mice. Age‐matched male and female C57BL/6J (Jackson Lab) were used as wild type (WT) controls. All animals were housed in a 12:12‐h light–dark cycle and provided with food (Teklad 2920x, Inotiv, Madison, WI) and water ad libitum. The study protocol was approved by the Institutional Animal Care and Use Committee at The Abigail Wexner Research Institute at Nationwide Children's Hospital, and all animals received humane care. Euthanasia when required was performed using intraperitoneal injections of ketamine (500 mg/kg)/xylazine (50 mg/kg).

### 2.2 Measurements

#### 2.2.1 Body weights

Body weights of male and female WT, 3A‐KO, and 3B‐KO mice (identified by genotyping at 7 days of life) were recorded weekly after birth using a digital scale to assess body weight trends. Date of birth, sex, and observed gross abnormalities were also recorded.

#### 2.2.2 Right ventricle (RV) assessments

The mice were anesthetized with intraperitoneal injections of xylazine/ketamine (4.5/90 mg/kg). A horizontal incision was made below the diaphragm and a pressure transducer was placed directly into the RV to measure right ventricular systolic pressures (RVSP) as previously described (Jin et al., 2010). Following the RV pressure measurements, a median sternotomy was performed, and the lungs removed, rinsed in ice‐cold PBS, pH 7.4, blotted dry, and snap frozen in liquid nitrogen for further analyses. The hearts were removed, rinsed in cold PBS, and weighed. The RV wall was carefully dissected from the left ventricle (LV) and septum (S). The RV and the LV + septum were weighed and the ratio of RV to (LV + S) weights (Fulton's index) was used to assess the degree of right ventricular hypertrophy (RVH). Tibial length was also measured to assess RV and LV mass, as determined by the RV weight normalized to tibial length and LV weight normalized to tibial length, respectively.

#### 2.2.3 Free Fatty Acid (FFA) measurements

Blood was drawn via tail vein collection from WT, 3A‐KO, and 3B‐KO mice. Serum was processed. Free FA levels were measured using a FFA quantitation kit (Sigma‐Aldrich, catalog #MAK044).

### 2.3 PASMC Isolation and Culture

Murine PASMC were isolated from four‐ to eight‐week‐old male (M) or female (F) 3A‐KO, 3B‐KO, or WT C57BL/6J (Jackson Lab) mice as previously described in detail by (Lee et al., 2013). The isolated PASMC (passage 3) were plated onto 6‐cm tissue culture plates and incubated in 21% O_2_–5% CO_2_ – balance N_2_ at 37°C. The cells were grown to 80%–90% confluence, and protein was isolated for Western blotting. Confirmation of PASMC was performed by verifying α‐SMA positive, PECAM‐1 negative and PDGFRα negative protein expression in the cells.

### 2.4 Pde3a or Pde3b knockdown using siRNA

Small interfering (si) RNA transfection was used to determine the effects of *Pde3a* and *Pde3b* gene silencing on downstream molecular pathways (Chen, Calvert, et al., 2009; Dillard et al., 2020). One day prior to transfection, PASMC were plated onto six‐well cell culture plates containing 2 mL of SMC growth media and grown to 70% confluence. For each well, 20 μL of 2 μM siRNA targeting against *Pde3a* (siPDE3A), *Pde3b* (siPDE3B), or non‐targeting scramble siRNA (scRNA, Santa Cruz Biotechnology, Santa Cruz CA, catalog #sc‐41,593, sc‐41,595, and sc‐37,007, respectively) was added to 0.5 mL of basal serum‐free SMC media (Cell Biologics Inc, Chicago, IL) and gently mixed. Lipofectamine 2000 (10 μL, Thermo Fisher, catalog #11668019) was diluted in 0.5 mL of basal serum‐free SMC media and incubated for 5 min at room temperature. The diluted siRNA and Lipofectamine 2000 mixture were combined and incubated for 20 min at room temperature. The siRNA/Lipofectamine mixture was added to each well (corresponding scRNA, siPDE3A, and/or siPDE3B) with an additional 1 mL of antibiotic‐free SMC media containing 2 μL each of included growth factors and 200 μL of FBS. The cells were incubated with the siRNA complex at 37°C for 24 h prior to the change to standard SMC growth media. Cell culture plates were subsequently incubated at 37°C for 24 h. The growth media was then changed, and the cells were incubated for an additional 24 h prior to protein harvest.

### 2.5 Protein Isolation

Protein concentrations in the soluble lysates were determined as previously described (Chen et al., 2012, 2014; Chen, Calvert, et al., 2009; Dillard et al., 2020). Briefly, PASMC were washed with PBS, and 60–80 μL of lysis buffer [HEPES (pH 7.4), β‐glycerophosphate, EGTA, DTT, NaF, Na_3_VO_4_, Triton X‐100 and glycerol] and protease inhibitors (leupeptin, aprotinin and PMSF) were added to 35‐mm plate or each well of a six‐well plate. The PASMC were scraped, pipetted into sterile centrifuge tubes, and placed on ice for 30 min. The cell lysate‐containing tubes were centrifuged at 4°C, 12,000 × *g* for 10 min. The pellet was discarded and the supernatant was stored in 1.5‐mL tubes at −80°C. Total protein concentration was determined via Bradford method (Bradford, 1976).

### 2.6 Western Blot

Harvested PASMC protein was assayed for PDE3A, PDE3B, α‐smooth muscle actin (α‐SMA), total and phosphorylated AMPK, total and phosphorylated cAMP response element‐binding protein (CREB), total and cleaved caspase 3, peroxisome proliferator activated receptor γ (PPARγ), PPARγ coactivator‐1α (PGC‐1α), pyruvate dehydrogenase kinase (PDK) 1–4, and β‐actin as previously described (Chen et al., 2012, 2014; Chen, Calvert, et al., 2009; Dillard et al., 2020). Blots were incubated in primary antibodies overnight, and then washed with PBS containing 0.1% Tween‐20 (PBST) three times. The blots were then incubated with the appropriate horseradish peroxidase (HRP)‐conjugated secondary antibody at room temperature for 1 h. After washing, the protein bands were visualized using enhanced chemiluminescence (Thermo Fisher, catalog #32106) and quantified using densitometry. To control for protein loading, the blots were stripped using a stripping buffer containing 62.5 mM Tris HCl (pH 6.8), 2% SDS, and 100 mM 2‐β‐mercaptoethanol, and the blots were reprobed for β‐actin (1:10,000, Millipore, catalog #37005) as described above. The following antibodies were used in Western blot analyses: PDE3A (1:500, Santa Cruz, catalog #sc‐293,446), PDE3B (1:250, Abcam, Cambridge, UK, catalog #ab95814), α‐SMA (1:10,000, Abcam, catalog #ab124964), total AMPK (1:250, ProteinTech, San Diego, CA, catalog #10929‐2‐AP), phosphorylated AMPK (p‐AMPK) (1:1000, Cell Signaling Technology, Danvers, MA, catalog #2535 T), total CREB (1:1000, Cell Signaling, catalog #9197S), phosphorylated CREB (p‐CREB) (1:1000, Cell Signaling, catalog #9198S), total caspase 3 (1:1000, Cell Signaling, catalog #9662), cleaved caspase 3 (1:1000, Cell Signaling, catalog #9664P), PPARγ (1:1000, Santa Cruz, catalog #sc‐7273), PGC‐1α (1:1000, Abcam, catalog #ab191838), PDK1 (1:100, ABclonal Technology, Woburn, MA, catalog #A0834), PDK2 (1:1000, ABclonal, catalog #A4737), PDK3 (1:1000, ABclonal, catalog #A8028), PDK4 (1:1000, ABclonal, catalog #A13337), HRP‐conjugated goat anti‐rabbit IgG (1:10,000, Boster, catalog #BST15L14B54), HRP‐conjugated goat anti‐mouse IgG (1:10,000, Bio‐Rad Laboratories, Hercules, CA, catalog #L006326), and HRP‐conjugated goat anti‐rat IgG (1:10,000, Bio‐Rad, catalog #170320). Representative Western blots are shown, and full unedited images are available at https://doi.org/10.6084/m9.figshare.27170658.

### 2.7 Adipocyte Isolation, siRNA Transfection and Free Fatty Acid Assay

Pre‐adipocytes were isolated from white adipose tissue using a preadipocyte isolation kit (Abcam, catalog #ab196988) and differentiated into mature adipocytes. Briefly, fresh adipose tissue was harvested and minced using sterile scissors. The minced adipose tissues were then mixed with collagenase B (Thermo Fisher, catalog #17104019) to achieve a final concentration of 6.6 mg/g adipose tissue. The mixture was incubated in a heated orbital shaker at 37°C for 30 min at 160 rpm. Collagenase stop buffer (Thermo Fisher) was added into the digestion at a ratio of 18 mL per g adipose tissue, and then mixed by inverting. The fat cell suspension was subsequently filtered through a 100‐μm cell strainer, and the filtrate was centrifuged at 500 x g for 10 min. The supernatant was removed, and the pellet was resuspended in 1 mL of Red Blood Cell lysis buffer for 1 min. PBS (9 mL) was added, and the cells were filtered through a 70‐μm cell strainer. The filtrate was centrifuged at 500 × *g* for 10 min before the supernatant was removed and pellet resuspended in 2 mL of pre‐adipocyte media (DMEM/F12, 10% FBS, P/S, amphotericin B). Cells were added into one well of a sterile six‐well cell culture plate and then incubated at 37°C for 12 h before changing media. Cells were grown until 80% confluence before differentiation into mature adipocytes using a 3 T3‐L1 differentiation kit (Abcam, catalog #ab287843) according to manufacturer's protocol. Mature adipocytes were transfected with scRNA, siPDE3A, or siPDE3B as described above and incubated at 37°C for 24 h. Adipocytes were washed with PBS, and fresh adipocyte media (DMEM/F12 (1:1) with 10% FBS) was added. The cells were incubated at 37°C for 48 h, after which the media was collected for FFA measurements and conditioned media experiments. Free FA levels in the media were measured using a FFA quantitation kit (Sigma‐Aldrich, catalog #MAK044) according to the manufacturer's protocol.

### 2.8 Conditioned Media Experiment

Following the 24‐h transfection, scramble siRNA‐ or siPDE3A‐transfected WT PASMC were incubated in a 1:1 ratio of SMC growth media and media collected from siPDE3A‐transfected adipocytes (conditioned media) as described above, or in a 1:1 ratio of SMC growth media and media collected from scRNA‐transfected adipocytes (control media). Therefore, three groups were compared: (1) scRNA‐transfected PASMC + scRNA adipocyte control media, (2) scRNA‐transfected PASMC + siPDE3A adipocyte conditioned media, and (3) siPDE3A‐transfected PASMC + scRNA control media. The cells were incubated with the control or conditioned media at 37°C for 48 h, and protein was harvested for Western blot analyses.

### 2.9 Viable Cell Numbers

We determined the effect of direct knockout of *Pde3a* in PASMC and the exposure of PASMC to bioactive mediators released into the media after siPDE3A transfection of adipocytes on PASMC growth. Briefly, scramble siRNA‐ and siPDE3A‐transfected PASMC were seeded at a density of 12.5 × 10^4^ cells per well of six‐well tissue culture plates and grown for a total of 7 days in either scRNA‐transfected control media or siPDE3A‐transfected conditioned media. On Days 1–7, one well of each group was washed twice with PBS and 0.5 mL of 0.25% trypsin was applied. Viable cells were counted manually via trypan blue exclusion as previously described (Chen et al., 2012; Chen, Calvert, et al., 2009).

### 2.10 Statistical Analysis

All studies were performed in duplicate or triplicate, where ‘n’ is the number of biological replicates from at least three animals per gender or the number of animals used for each experiment. Male and female cells were analyzed and pooled if no statistical significance was observed between genders. In the figures, *n* represents the total number of sample data points for a given experiment. Values are presented as means ± SD. Unpaired Student's *t*‐test, one‐way or two‐way ANOVA with post hoc Tukey's analyses for multiple comparisons were used to compare groups as appropriate (GraphPad Prism Software, La Jolla, CA). Differences were considered significant when *p* < 0.05.

## 3 RESULTS

### 3.1 *Pde3* *a* knockout mice have lower body weights than *Pde3b* knockout and WT mice

**FIGURE 1:**
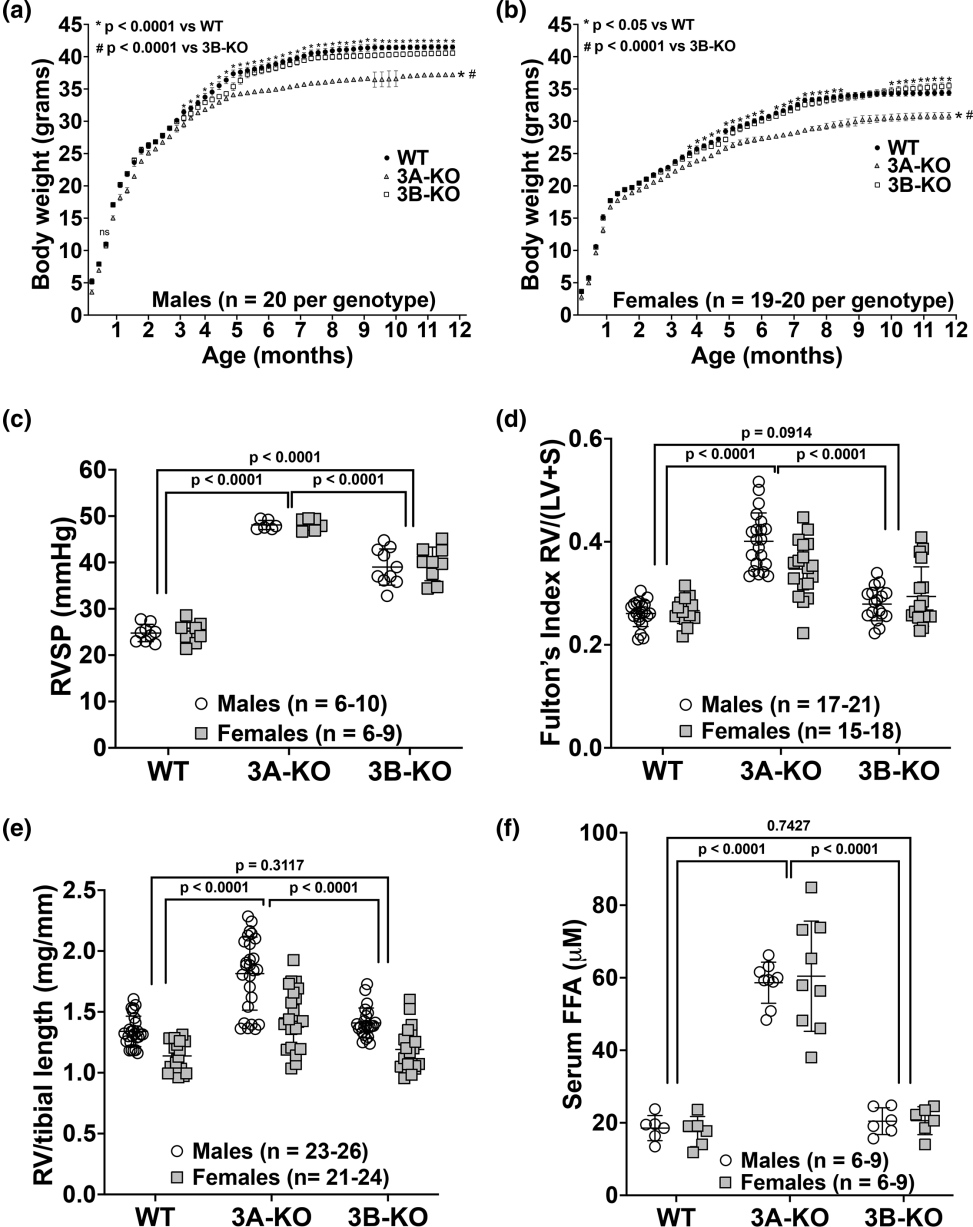
*Pde3a*‐deficient are smaller in size, have higher right ventricular (RV) systolic pressures (RVSP), severe RV hypertrophy (RVH), increased RV mass, and elevated serum free fatty acid (FFA) levels. Body weight, RV(LV + S) and RV/tibial length measurements in m male and female WT, 3A‐KO, and 3B‐KO mice. Body weights were measured weekly in WT, 3A‐KO, and 3B‐KO mice from birth to 12 months of age. Body weights (grams) in male (a) and female (b) mice. RVSP (mmHg) was measured in five‐month‐old mice (c), Fulton's index [RV/(LV + S)] was calculated to assess RVH in mice one to five‐months of age (d), RV/tibial length (mg/mm) was calculated to assess RV mass (e). Serum was processed from tail vein blood collection and FFA levels (μM) were measured (f).

Adult WT, 3A‐KO, and 3B‐KO mice were maintained in room air without any exposures or treatments. Both male and female 3A‐KO were markedly smaller and noted to have significantly lower body weight trends at nearly every weekly time point than age‐ and sex – matched male (Figure 1a) and female (Figure 1b) WT and 3B‐KO mice from one to 12 months of life. Compared to WT mice, 3B‐KO mice were noted to have statistically significant differences in body weights; however, less obvious than with 3A‐KO mice. Interestingly, male 3B‐KO mice had lower body weights than WT mice from 3 to 12 months of age (Figure 1a), whereas in females lower body weights were observed between 4 and 8 months and higher body weights at 10–12 months of age compared to WT mice (Figure 1b).

### 3.2 *Pde3a*‐knockout mice exhibit elevated right ventricular systolic pressures

Wild‐type, 3A‐KO, and 3B‐KO mice (5 months of age) were maintained in room air without any exposures or treatments. Right ventricular systolic pressures were measured via insertion of a pressure transducer directly into the RV of each mouse. WT mice had an average RVSP of 24.8 mmHg ±2.0 SD. The RVSP of 3A‐KO (48.2 mmHg ±1.1 SD) and 3B‐KO (39.3 mmHg ±3.7 SD) were both elevated compared to the WT, *p* < 0.0001 (Figure 1c). Furthermore, 3A‐KO mice had significantly higher RVSP than 3B‐KO mice (*p* < 0.0001, Figure 1c). There were no differences in RVSP between genders in any of the groups.

### 3.3 *Pde3a*‐knockout mice develop right ventricular hypertrophy

We assessed Fulton's index in WT, 3A‐KO, and 3B‐KO (1–5 months of age) mice to determine the effect of elevated PA pressures on the RV. Right ventricular hypertrophy was evident in the 3A‐KO mice relative to the WT and 3B‐KO mice (Figure 1d, Fulton's index 0.37 ± 0.06 SD (3A‐KO) vs. 0.26 (WT) ± 0.02 SD and 0.29 (3B‐KO) ± 0.05 SD, *p* < 0.0001). There were no statistically significant differences in RVH between WT and 3B‐KO mice (*p* = 0.0914). RV mass was also determined by the RV weight (mg) normalized to tibial length (mm) as an additional assessment. Similar to Fulton's index, 3A‐KO mice had a significantly greater RV mass than WT and 3B‐KO mice [Figure 1e, 1.72 mg/mm (3A‐KO) ± 0.27 SD vs. 1.33 mg/mm (WT) ± 0.12 SD and 1.42 mg/mm (3B‐KO) ± 0.14 SD, *p* < 0.0001]. There were no differences in RV mass between WT and 3B‐KO mice. There were differences in Fulton's index between 3A‐KO males and females, as well as in RV mass between genders for each genotype; however, trends between genotypes remained consistent for both males and females.

### 3.4 *Pde3a*‐deficient mice have elevated serum FFA levels

Serum FFA levels were significantly higher in 3A‐KO mice than in WT and 3B‐KO mice [Figure 1f, 59.5 μM (3A‐KO) ± 11.2 vs. 18.1 μM (WT) ± 3.7 and 20.5 μM (3B‐KO) ± 3.6 SD, *p* < 0.0001]. There were no differences in serum FFA between genders in any of the groups.

### 3.5 *Pde3a* deficiency causes no compensatory changes in PDE3B PASMC protein expression, whereas *Pde3b* knockdown by siRNA leads to a decrease in PDE3A protein expression

**FIGURE 2:**
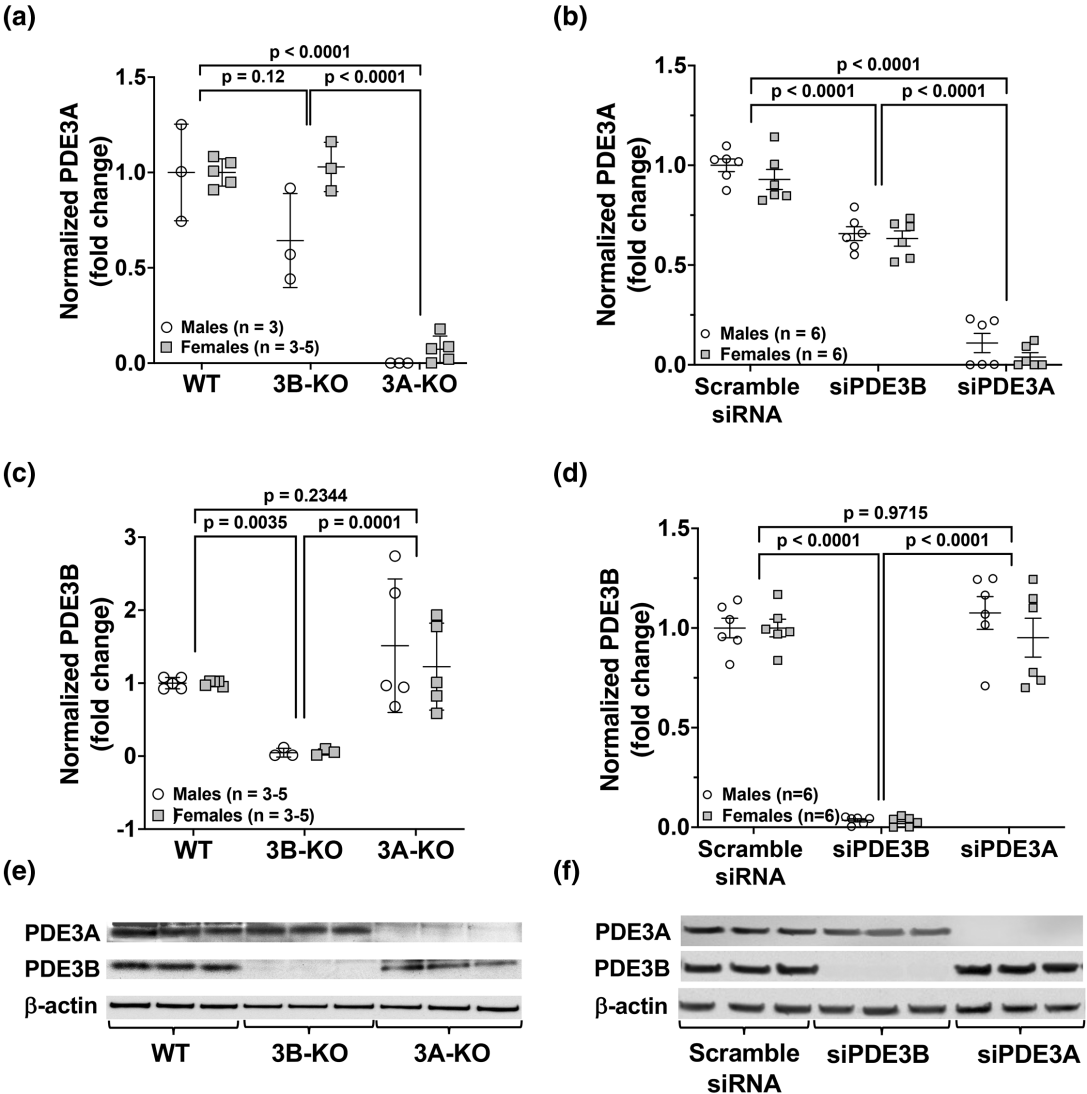
*Pde3a* deficiency does not lead to compensatory changes in PDE3B protein expression, whereas *Pde3b* knockdown by siRNA leads to a decrease in PDE3A protein expression. Isolated PASMC from male and female WT, 3A‐KO and 3B‐KO mice were cultured and grown at 37°C until 80%–90% confluent or isolated PASMC from WT mice were transiently transfected with scramble siRNA, siRNA targeted against *P* *de3a* (siPDE3A), or *P* *de3b* (siPDE3B) and incubated at 37°C for 24 h. Protein was harvested and PDE3A (a, b) and PDE3B (c, d) were analyzed by Western blot and densitometry data was normalized to loading control and expressed as fold change ± SD. Representative Western blots are shown (e, f), and full images are found in the supplementary data.

To confirm the deficiency of PASMC PDE3A and PDE3B protein, PASMC were isolated from male and female 3A‐KO and 3B‐KO mice or PASMC were isolated from WT animals, which were then transiently transfected with scRNA, siPDE3A or siPDE3B, and cultured at 37°C. We utilized siRNA techniques to knockdown *Pde3* subtypes in WT PASMC, rather than isolated KO cells to allow for greater control of conditions. Because the genetically‐modified *Pde3a and Pde3b* mice are global knockouts, the effect of knockout of *Pde3* subtypes in other tissues could potentially have effects on PASMCs. Utilizing siRNA against subtype‐specific *Pde3* allows the study of the effect of the single gene deficiency on varying metabolic pathways. Protein lysate was isolated and analyzed by Western blot. Western blot analyses confirmed the absence of PDE3A in PASMC isolated from 3A‐KO and siPDE3A‐transfected WT PASMC (Figure 2a,b,e,f) and the absence of PDE3B in PASMC isolated from 3B‐KO mice and siPDE3B‐transfected PASMC (Figure 2c–f). There were no compensatory changes observed in PDE3A protein expression in the 3B‐KO PASMC (Figure 2a); however, siRNA knockdown of *P* *de3b* decreased PDE3A protein expression (Figure 2b). There was no change in PDE3B protein expression in the 3A‐KO PASMC (Figure 2c) or with siRNA knockdown of *P* *de3a* in WT PASMC (Figure 2d). No gender differences were observed.

### 3.6 *Pde3a* deficiency in PASMC leads to robust changes in key proteins involved in cellular proliferation, metabolism, and apoptosis

**FIGURE 3:**
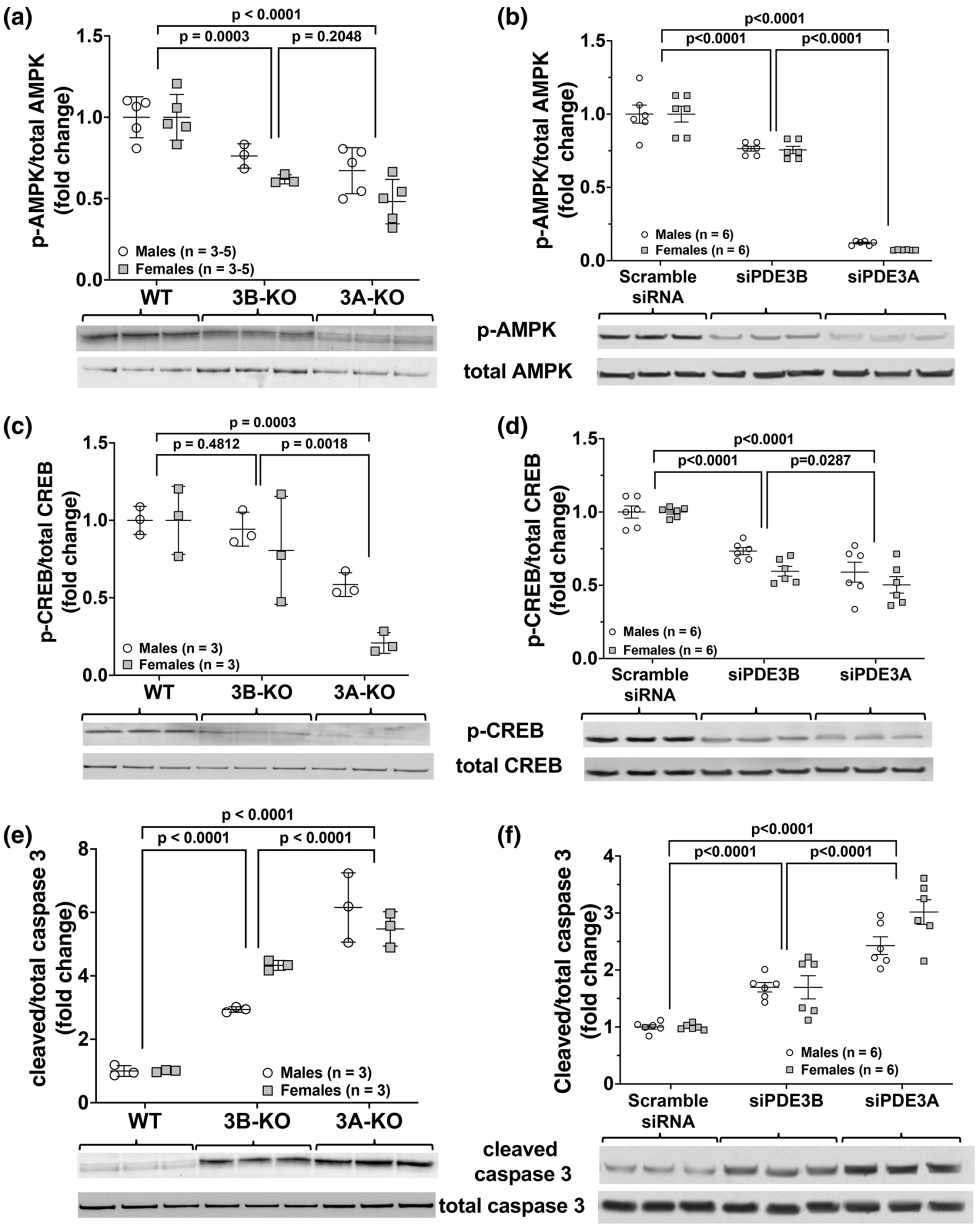
*Pde3a* deficiency in PASMC leads to more robust decreases in AMPK and CREB phosphorylation and increases in cleaved caspase 3 protein expression than *Pde3b* deficiency. Isolated PASMC from male and female WT, 3A‐KO and 3B‐KO mice were cultured and grown at 37°C until 80%–90% confluent or isolated PASMC from WT mice were transiently transfected with scramble siRNA, siRNA targeted against *P* *de3a* (siPDE3A), or *P* *de3b* (siPDE3B) and incubated at 37°C for 24 h. Protein was harvested and phosphorylated (p)‐ and total AMPK (a, b), p‐ and total CREB (c, d), and cleaved and total caspase 3 (e, f) were analyzed by Western blot and densitometry data was normalized to loading control and is shown as fold change ± SD. Representative Western blots are shown, and full images are found in the supplementary data.

PDE3 has been shown to regulate signaling pathways related to proliferation and metabolism, including AMPK and CREB, as well as apoptosis (Chung et al., 2017; Dillard et al., 2020; Ding et al., 2005; Liu et al., 2006). To determine the effects of PDE3A and PDE3B deficiency on these pathways, protein lysate was harvested and analyzed for activation of AMPK, CREB and caspase 3. We found that AMPK activity was significantly lower in *Pde3a*‐deficient PASMC compared to WT PASMC controls (*p* < 0.0001, Figure 3a,b). Likewise, there was a significant decrease in CREB phosphorylation in *Pde3a*‐deficient PASMC compared to WT PASMC controls (*p* ≤ 0.0003, Figure 3c,d). *Pde3b* deficiency led to less robust effects on AMPK and CREB phosphorylation, with an overall decrease in AMPK and CREB phosphorylation compared to WT PASMC, but less prominent than the effects observed from deficiency in *Pde3a* (Figure 3).

Interestingly, there was a substantial rise in activated caspase 3 in both 3A‐KO and siPDE3A‐transfected WT PASMC (*p* < 0.0001) relative to WT controls (Figure 3e,f). Although *Pde3b* deficiency resulted in a rise in activated caspase 3, the increase was significantly less than observed in *Pde3a*‐deficient PASMC (Figure 3e,f).

**FIGURE 4:**
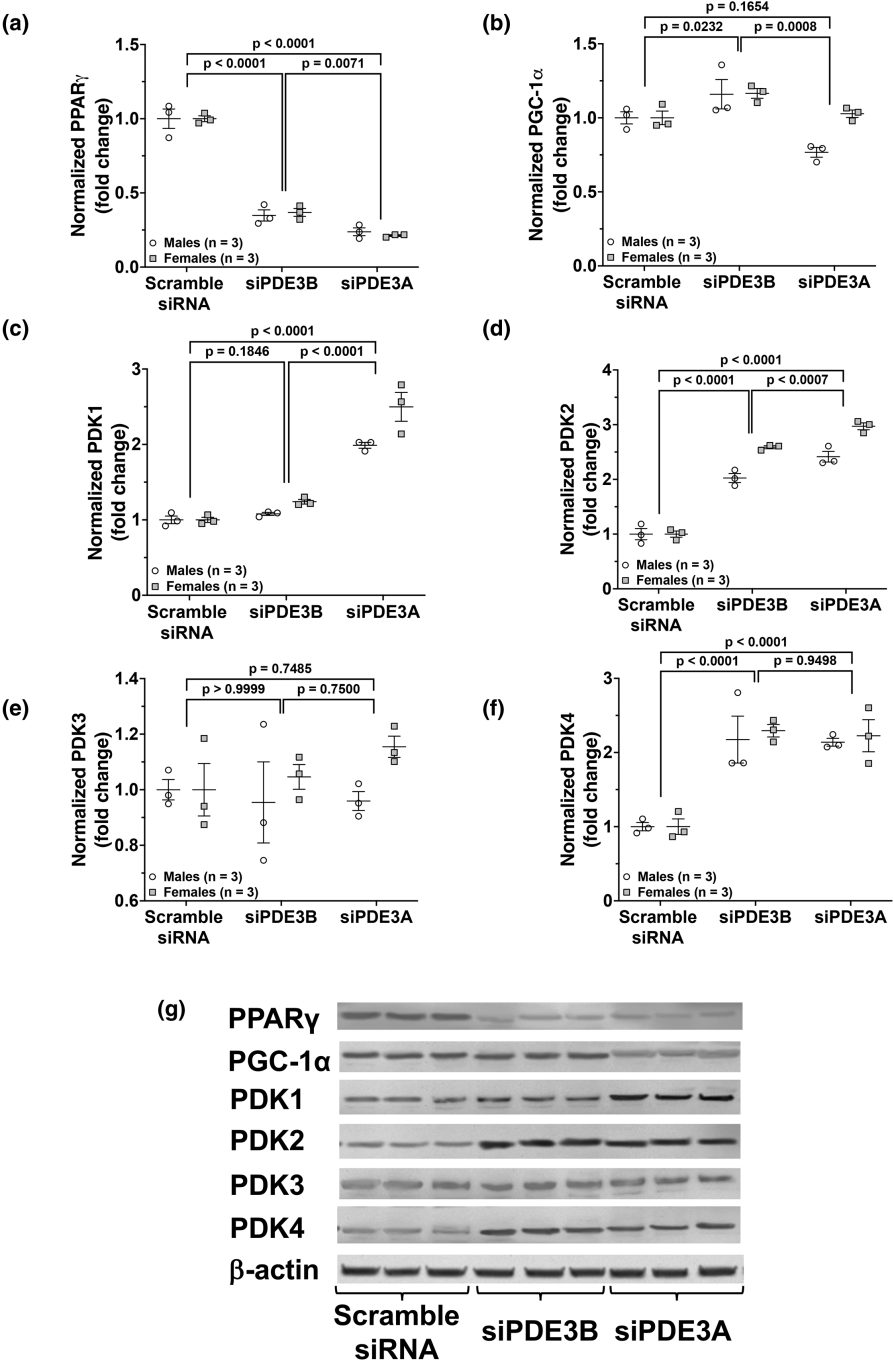
*Pde3a* knockdown in PASMC by siRNA leads to more robust decreases in PPARγ and increases in pyruvate kinase dehydrogenase (PDK) protein expression than *Pde3b* knockdown. Isolated PASMC from WT mice were transiently transfected with scramble siRNA, siRNA targeted against *P* *de3a* (siPDE3A), or *P* *de3b* (siPDE3B) and incubated at 37°C for 24 h. Protein was harvested and PPARγ (a), PGC‐1α (b), PDK1 (c), PDK2 (d), PDK3 (e), PDK4 (f) were analyzed by Western blot and normalized to loading control, and densitometry data are shown as fold change ± SD (a, b) or normalized data (arbitrary units) ± SD (c–e). Representative Western blots are shown (g), and full images are found in the supplementary data.

The effects of *Pde3a* and *Pde3b* knockdown using siRNA on the protein expression of PPARγ and PGC‐1α were evaluated. We found that compared to scRNA‐transfected PASMC, PPARγ levels were significantly decreased in siPDE3A‐transfected and siPDE3B‐transfected WT PASMC (*p* < 0.0001, Figure 4a,g). We found that levels of PGC‐1α, a master regulator of mitochondrial biogenesis that is downstream of AMPK and a critical cofactor for PPARγ, were modestly increased in siPDE3B‐transfected PASMC and unchanged in siPDE3A‐transfected PASMC (Figure 4b,g).

Several studies have provided evidence to support the notion that metabolic abnormalities, such as a shift from mitochondrial oxidative phosphorylation to aerobic glycolysis, contribute to the proliferative phenotype of PASMC in PH (Lechartier et al., 2022; Paulin & Michelakis, 2014; Ryan et al., 2015). This metabolic shift has been attributed to inhibition of the pyruvate dehydrogenase complex (PDC), which controls the final step of the conversion of pyruvate to acetyl‐CoA for the TCA cycle, by PDKs (Lechartier et al., 2022; Michelakis et al., 2017; Ryan et al., 2015). Thus, inhibition of PDC by PDKs results in less substrate produced for mitochondrial oxidative phosphorylation. Therefore, the effects of *Pde3a* and *Pde3b* knockdown using siRNA on the protein expression of PDK1‐4 were also evaluated. We found robust increases in PDK1, PDK2 and PDK4 following knockdown of *Pde3a*, and significant increases in PDK2 and PDK4 after knockdown of *Pde3b* in PASMC (Figure 4c–g).

### 3.7 *Pde3a* knockdown in adipocytes results in release of FFA into the media

**FIGURE 5:**
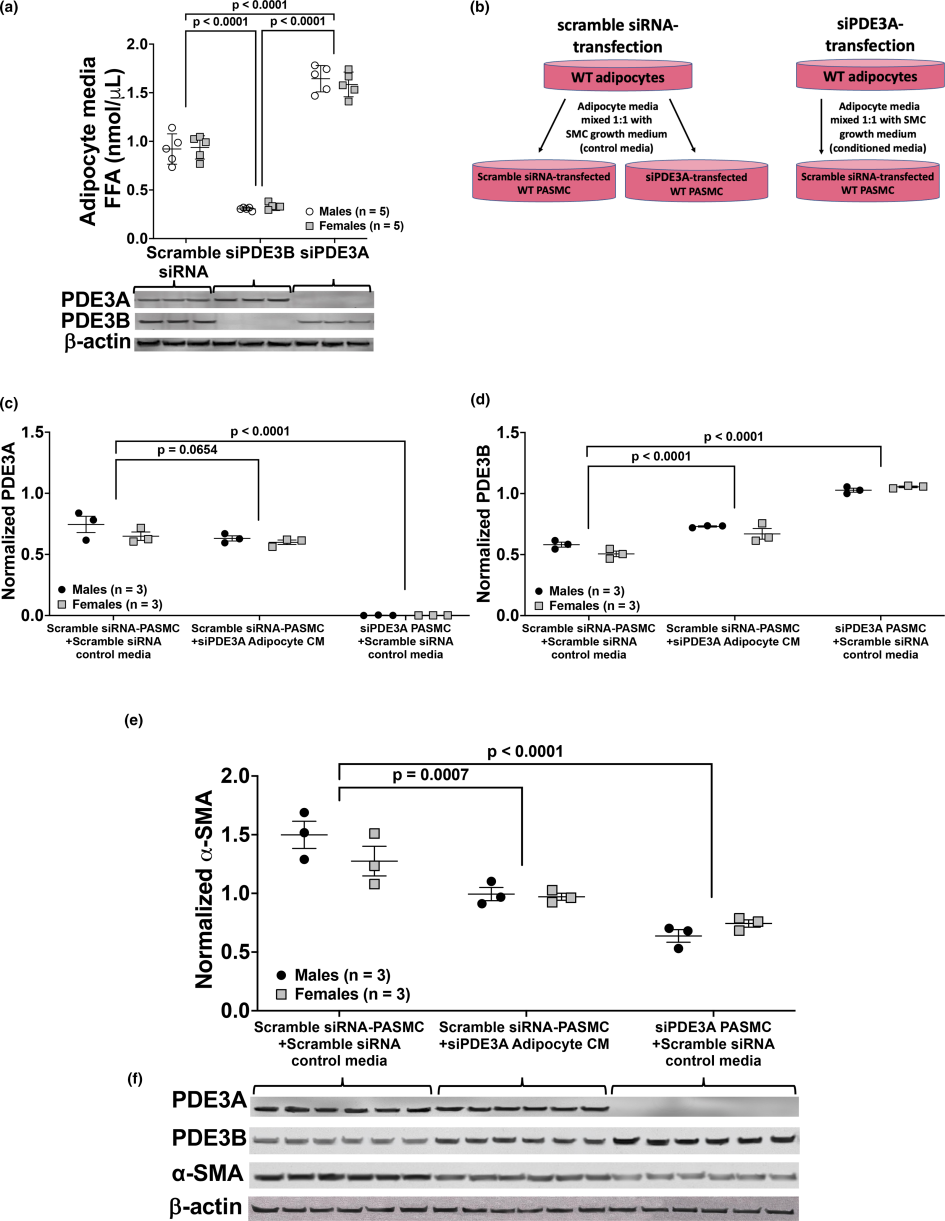
FFA levels are higher in media collected from adipocytes transiently transfected with siRNA targeted against *P* *de3a* (siPDE3A). Exposure of siPDE3A‐transfected PASMC to adipocyte control media and exposure of scramble siRNA‐transfected WT PASMC to adipocyte conditioned media (CM) that have high free fatty acid (FFA) levels lead to an increase in PDE3B and decrease in α‐SMA protein expression. Scramble siRNA (scRNA), siPDE3A, or siPDE3B was transiently transfected into adipocytes isolated from male and female WT mice. Media was collected after 48 h for FFA quantitation (a) and used as conditioned media. Representative Western blot below showing adequate knockdown of PDE3B and PDE3A, and full images are found in the supplementary data. Schematic of conditioned media (CM) experiment (b): Transfected PASMC with scRNA or siPDE3A were incubated at 37°C for 48 h in a 1:1 ratio of SMC growth media and either (1) media collected from siPDE3A‐transfected adipocytes (CM), or (2) media collected from scRNA‐transfected adipocytes (control media). Protein was harvested for Western blot analyses. PDE3A (c), PDE3B (d) and α‐SMA (e) were analyzed by Western blot and normalized to loading control and densitometry data are shown as normalized data (arbitrary units) ± SD. Representative Western blots are shown (f), and full images are found in the supplementary data.

Our results demonstrate a potential role for PDE3A in adipocyte lipolysis with lower body weights (Figure 1a,b) and increased serum FFA levels in 3A‐KO mice (Figure 1f) (Michelakis et al., 2017); therefore, we hypothesized that deficiency of *Pde3a* leads to excessive FFA production that may be contributing to altered metabolic processes in other cell types such as PASMC. Consequently, we measured the FFA content in the cell culture media following subtype‐specific *Pde3* knockdown in adipocytes using a commercially available assay kit. Subtype‐selective knockdown was confirmed by Western blot (Figure 5a). Adipocytes isolated from WT mice that were transfected with siPDE3A were found to have significantly higher levels of FFA released into the media when compared to FFA levels in scRNA‐ and siPDE3B‐transfected adipocyte media (*p* < 0.0001, Figure 5a). Conversely, *Pde3b* knockdown in WT adipocytes resulted in significantly lower FFA levels in the media when compared to levels in the media of scRNA‐transfected WT adipocytes (*p* < 0.0001, Figure 5a).

### 3.8 *Pde3a*‐deficient PASMC incubated in adipocyte media and scRNA‐transfected WT PASMC incubated in siPDE3A‐transfected adipocyte media with high FFA content induce PDE3B and inhibit α‐SMA protein expression

Experiments were performed to determine whether exposure of WT PASMC to the high FFA levels within the siPDE3A‐transfected adipocyte media results in differential effects on PDE3A, PDE3B and α‐SMA protein levels as compared to the effects from direct *Pde3a* knockdown in the WT PASMC. We hypothesized that exposure to either the elevated FFA or other lipotoxic metabolites results in metabolic pathway changes in PASMC. Therefore, conditioned media using siPDE3A‐transfected adipocyte media mixed 1:1 with SMC media was added to scRNA‐transfected PASMC, whereas control media from scRNA‐transfected adipocytes mixed 1:1 with SMC media was added to scRNA‐transfected PASMC or siPDE3A‐transfected PASMC (Figure 5b) and incubated at 37°C for 48 h. Protein was harvested and expression for PDE3A, PDE3B, α‐SMA and β‐actin were quantified by Western blot. As expected, PDE3A was knocked down in the siPDE3A‐transfected PASMC + scRNA adipocyte control media (Figure 5c,e). PDE3A protein levels were not different between the scRNA‐transfected PASMC + siPDE3A‐transfected adipocyte conditioned media compared to those scRNA‐transfected PASMC + scRNA‐transfected adipocyte control media (Figure 5c,f). Conversely, PDE3B protein expression was significantly higher with direct PASMC *Pde3a* knockdown compared to scRNA‐transfected PASMC + scRNA‐transfected adipocyte control media (Figure 5d,f). In addition, there was a less robust but statistically significant increase in PDE3B protein expression in the scRNA‐transfected PASMC + siPDE3A‐transfected adipocyte conditioned media compared to scRNA‐transfected PASMC + scRNA‐transfected adipocyte control media (*p* < 0.0001, Figure 5d,f). Alpha‐SMA protein expression was significantly lower with direct PASMC *Pde3a* knockdown compared to scRNA‐transfected PASMC + scRNA‐transfected adipocyte control media (Figure 5e,f). There was also a less substantial but statistically significant decrease in α‐SMA protein expression in the scRNA‐transfected PASMC + siPDE3A‐transfected adipocyte conditioned media compared to exposure to scRNA‐transfected PASMC + scRNA‐transfected adipocyte control media (*p* < 0.001, Figure 5e,f).

### 3.9 Exposure of PASMC to high FFA content inhibits AMPK and CREB phosphorylation and increases activated caspase 3 expression

**FIGURE 6:**
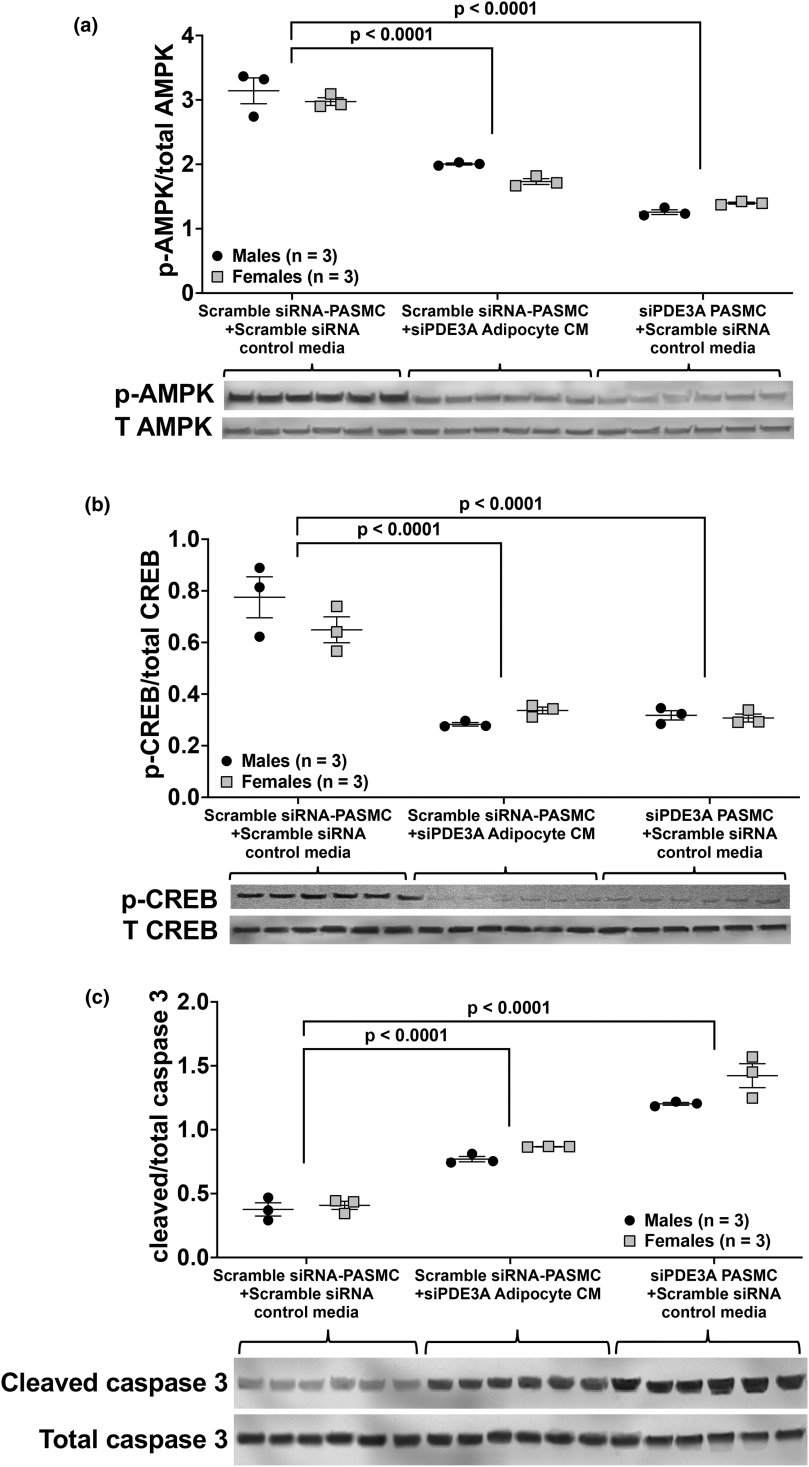
Exposure of scramble siRNA‐transfected WT PASMC to conditioned media (CM) from adipocytes transfected with siRNA targeted against *P* *de3a* (siPDE3A) leads to similar but less robust decreases in AMPK and CREB phosphorylation and increases in caspase 3 activity than direct knockdown of PASMC *Pde3a*. Scramble siRNA (scRNA) or siPDE3A were transiently transfected into adipocytes isolated from male and female WT mice. Media was collected after 48 h and used as conditioned media. Transfected PASMC with scRNA or siPDE3A were incubated at 37°C for 48 h in a 1:1 ratio of SMC growth media and either (1) media collected from siPDE3A‐transfected adipocytes (CM), or (2) media collected from scRNA‐transfected adipocytes (control media). Protein was harvested for Western blot analyses. Phosphorylated (p)‐ and total AMPK (a), CREB (b), and total and cleaved caspase 3 (c) were analyzed by Western blot and normalized to loading control, and densitometry data are shown as normalized data (arbitrary units) ± SD. Representative Western blots are shown, and full images are found in the supplementary data.

Utilizing the previously described conditioned media experiment, p‐AMPK, T AMPK, p‐CREB, T CREB, cleaved and T caspase 3, and β‐actin were quantified by Western blot. Overall, we found that culture of scRNA‐transfected PASMC in siPDE3A‐transfected adipocyte conditioned media resulted in similar trends in protein expression as the direct knockdown of *Pde3a* in PASMC. Culture of siPDE3A‐transfected PASMC in scRNA‐transfected adipocyte control media resulted in significantly lower AMPK activity compared to scRNA‐transfected PASMC cultured in scRNA‐transfected adipocyte control media (*p* < 0.0001, Figure 6a). Culture of scramble siRNA‐transfected PASMC in siPDE3A‐transfected adipocyte conditioned media also resulted in lower AMPK activity compared to scRNA‐transfected PASMC cultured in scRNA‐transfected adipocyte control media (*p* < 0.0001, Figure 6a). Compared to scRNA‐transfected PASMC cultured in scRNA‐transfected adipocyte control media, CREB phosphorylation was similarly decreased with both the direct PASMC *Pde3a* knockdown and in scRNA‐transfected PASMC cultured in siPDE3A‐transfected adipocyte conditioned media (*p* < 0.0001, Figure 6b). Similar to previous results (Figure 3e,f), cleaved caspase 3 was significantly increased in the siPDE3A‐transfected PASMC cultured in scRNA‐transfected adipocyte control media compared to scRNA‐transfected PASMC in the same media, while scRNA‐transfected PASMC cultured in siPDE3A adipocyte conditioned media had less of an effect on caspase 3 activity (*p* < 0.0001, Figure 6c).

### 3.10 *Pde3a*‐deficient PASMC exposed to adipocyte media and scramble siRNA‐transfected PASMC exposed to siPDE3A‐transfected adipocyte media with high FFA content increase PASMC viability

**FIGURE 7:**
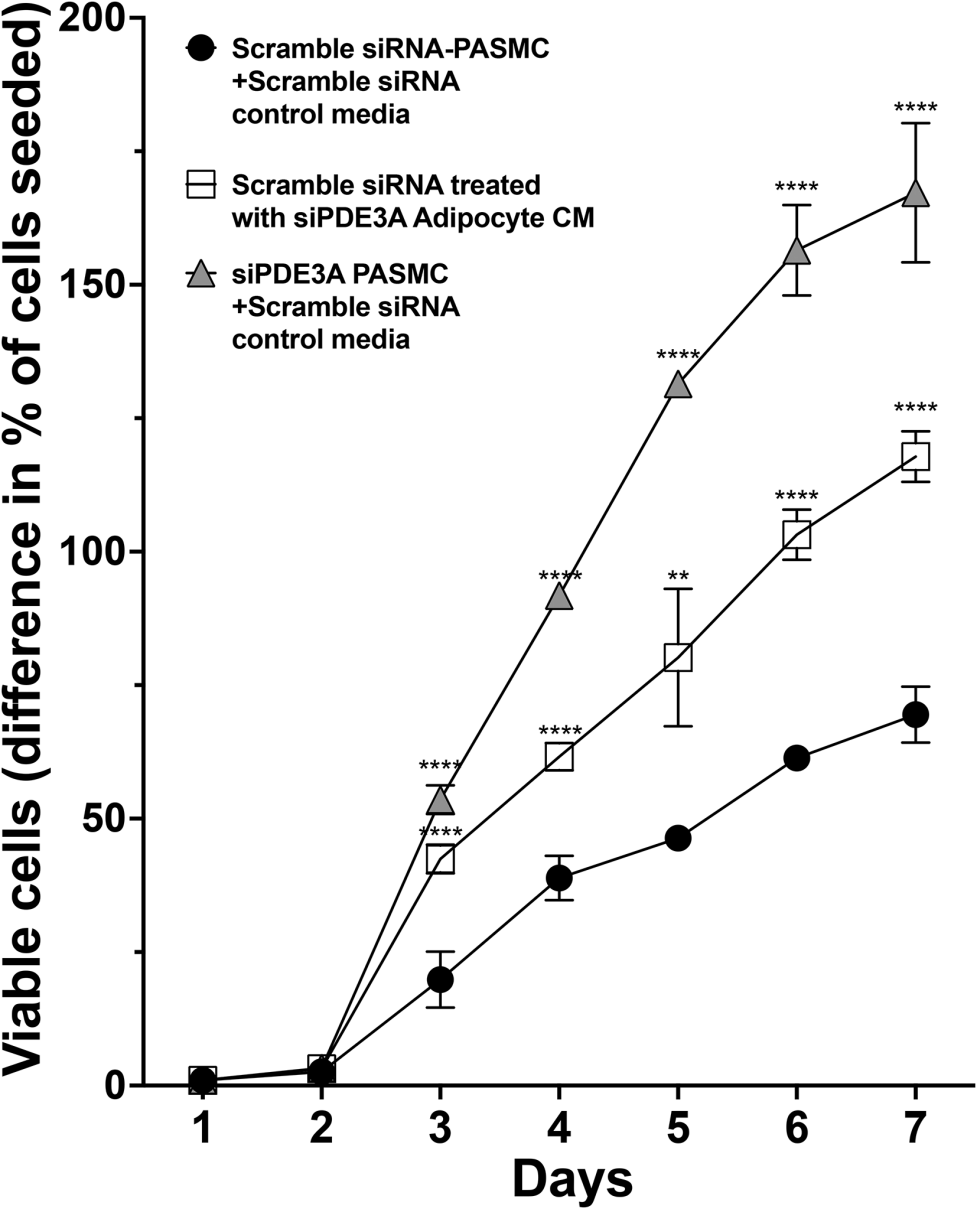
Exposure of scramble siRNA‐transfected WT PASMC to conditioned media (CM) from adipocytes transfected with siRNA targeted against *P* *de3a* (siPDE3A) increases viable cell numbers, similar to but less robust than observed with direct knockdown of PASMC *Pde3a*. Scramble siRNA or siPDE3A was transiently transfected into adipocytes isolated from male and female WT mice. Media was collected after 48 h and used as conditioned media. Scramble siRNA‐transfected adipocyte media mixed (1:1) with SMC growth media (control media) was added to scramble siRNA or siPDE3A‐transfected PASMC, or CM was added to scramble siRNA‐transfected WT PASMC for 7 days. Viable cell numbers were counted daily using trypan blue exclusion. **p < 0.01, ****p < 0.0001 versus scramble siRNA‐PASMC+scramble siRNA control media.

Finally, to determine cell viability, PASMC were seeded in SMC culture media and grown for 7 days. Viable cell numbers were manually counted daily via trypan blue exclusion. Three cell types were evaluated (Figure 5b): (1) scRNA‐transfected PASMC + scRNA‐transfected adipocyte control media, (2) scRNA‐transfected PASMC + siPDE3A‐transfected adipocyte conditioned media, and (3) siPDE3A‐transfected PASMC + scRNA‐transfected adipocyte control media. After 7 days, scRNA‐transfected PASMC had the lowest number of viable cells. Scramble siRNA‐transfected PASMC + siPDE3A adipocyte conditioned media and siPDE3A‐transfected PASMC demonstrated increased viable cell numbers with the latter demonstrating the greatest increase in viable cell numbers (Figure 7).

## 4 DISCUSSION

The two PDE3 subtypes, PDE3A and PDE3B, exhibit differing patterns of expression in various cell types (Dillard et al., 2020), with both subtypes expressed in PASMC (Dillard et al., 2020; Lorigo et al., 2021). PDE3A is highly expressed in platelets, airway, vascular smooth muscle, and cardiovascular tissues (Marshall et al., 2018). PDE3A has been postulated to be responsible for the regulation of systemic vascular SMC proliferation and is generally thought to be important in the regulation of cardiac and vascular smooth muscle contractility (Begum et al., 2011; Manganiello et al., 1995; Sun et al., 2007). PDE3B is more abundantly expressed in adipose tissue and is known to be the important subtype in regulating energy metabolism (Chung et al., 2017; Sun et al., 2007). However, recent data have shown that deletion of PDE3B may protect the murine cardiac tissue from ischemic reperfusion injury (Chung et al., 2015) and our present data demonstrate that PDE3A may play a significant role in energy metabolism. Thus, there is crossover in cellular/physiologic function of *Pde3* subtypes. The primary objective of this study was to explore the relationship between subtype‐specific PDE3 deficiencies and metabolic pathways related to cell growth and proliferation of PASMC. Although we found that *Pde3b* knockout mice show elevated trends in RVH and increased RVSP, *Pde3a* knockout mice exhibit more severe right ventricular hypertrophy, increased RV mass, and significant elevations in RVSP (Figure 1c,d). These findings were associated with evidence of metabolic disease including lower body weights (Figure 1a,b), increased adipose tissue lipolysis, skeletal muscle atrophy, increased serum FFA (Figure 1f), and hepatocellular injury (McGeorge et al., 2023). These findings support the hypothesis that deficiencies of *Pde3a* and *Pde3b* affect cellular metabolic pathways and that *Pde3a* deficiency may lead to a disruption of downstream cellular metabolic pathways mediated by elevated FFA levels or other lipotoxic metabolites.

Our present findings support the idea that *Pde3a* may play a significant role in maintaining homeostatic metabolic function. Metabolic theories have been suggested as an underlying etiology for PH, and metabolic derangements play a significant role in the pathogenesis of pulmonary vascular cell proliferation in PH (Canto & Auwerx, 2009; Culley & Chan, 2018; Marshall et al., 2018). There is also growing evidence for the role of mitochondrial dysfunction in pulmonary vascular cells that leads to impaired vascular relaxation, increased proliferation, and failure of regulatory mechanisms (Marshall et al., 2018; Zhang et al., 2022). Importantly, there is an unmet need to explore and understand the role of metabolic and mitochondrial dysfunction within the pulmonary vasculature (Humbert, Sitbon, & Simonneau, 2004). Current PH therapies are limited in function and serve as supportive treatment rather than curative therapy, lacking the ability to reverse cellular proliferation and metabolic dysfunction. Given the marked phenotype of significant right‐sided heart disease observed in the *Pde3a* knockout mice, our goal was to explore important central metabolic targets in PASMC that may be regulated by the *Pde3* subtypes.

AMPK is a protein kinase that acts as a sensor of cellular energy status (Rodriguez et al., 2021; Teng et al., 2013). It is activated in association with metabolic stress and these activation patterns are known to be altered in PH (Canto & Auwerx, 2009; Rodriguez et al., 2021). AMPK activation leads to increases in PGC‐1α expression, a master regulator of mitochondrial biogenesis, thereby leading to modulation of mitochondrial gene expression (Canto & Auwerx, 2009; Yeligar et al., 2018). Despite the variable effects of AMPK on PASMC proliferation, (Rodriguez et al., 2021; Wu et al., 2014) vascular cells have been shown to have decreased AMPK activity and PGC‐1α in models of PH (Canto & Auwerx, 2009; Teng et al., 2013). AMPK has also been found to be critical in the regulation of fatty acid metabolism, thermogenesis, and the development of adipose tissue. PDE3B has been shown to regulate AMPK activity (Chung et al., 2017). AMPK directly phosphorylates PGC‐1α and may therefore be a link between the sensing of energy status and the induction of transcriptional pathways that control energy expenditure (Canto & Auwerx, 2009). PDE3B knockout mice have increased AMPK activity, increased fatty acid oxidation and oxygen consumption (Chung et al., 2017; Wu et al., 2018). Furthermore, in adipocytes, PDE3B deficiency enhances cAMP/PKA signaling through CREB phosphorylation, as well as activation of AMPK, triggering the upregulation of critical mitochondrial proteins such as PGC‐1α (Chung et al., 2017). Our present experiments demonstrate that loss of either *Pde3a* or *Pde3b* led to a decrease in AMPK activation in PASMC. Given the findings of more robust changes in downstream targets, we speculate that in PASMC *Pde3a* may have greater regulatory control on AMPK than *Pde3b*. This notion is supported by our previous data demonstrating the regulation of AMPK by *Pde3a* in PASMC (Dillard et al., 2020). We reported that nitric oxide (NO) increases AMPK via PDE3A‐dependent mechanisms in human PASMC (hPASMC), whereas the same effect was not seen with PDE3B (Dillard et al., 2020). We found that deficiency of PDE3A blunted NO‐induced AMPK activation (Dillard et al., 2020). We speculate that in our previously published data (Dillard et al., 2020), the lack of any effect of *Pde3b* deficiency on AMPK levels may have been due to the incomplete knockdown of hPASMC PDE3B protein with siRNA transfection or differences in protein turnover rates. Nonetheless, our data supports the idea of the presence of differential regulatory mechanisms on AMPK activation by *Pde3* subtypes, which is likely to be cell type specific.

Activation of AMPK has been shown to be essential for the maintenance of PDC activity and the TCA cycle in cancer cells. Cai et al. (Cai et al., 2020) found that the AMPK‐mediated phosphorylation of PDC blocks the interaction of PDC with the PDC inhibitor PDK1, thereby resulting in maintenance of PDC activity and allowing for cancer cell metastasis (Cai et al., 2020). The Warburg Effect in cancer cells has been referenced as occurring in the proliferative phenotype of PASMC in PH (Lechartier et al., 2022; Paulin & Michelakis, 2014; Ryan et al., 2015). This phenomenon describes the metabolic shift in tumors from utilizing mitochondrial oxidative phosphorylation to aerobic glycolysis, where most of the glucose is fermented to lactate (Lechartier et al., 2022; Paulin & Michelakis, 2014; Ryan et al., 2015). We speculate that in *Pde3*‐deficient PASMC, the increase in PDKs may be due to the decreased activation of AMPK preventing the phosphorylation of the PDC and thus resulting in inhibition of PDC, the conversion of pyruvate to acetyl‐CoA, and oxidative phosphorylation.

PPARγ is a critical protein in mitochondrial gene expression and biogenesis (Teng et al., 2013), which we speculate may also contribute to the underlying metabolic etiologies of pulmonary vascular dysfunction by altering PASMC proliferation. PPARγ is a ligand‐activated transcription factor that regulates cell metabolism and proliferation (Canto & Auwerx, 2009; Yeligar et al., 2018). In PASMC, PPARγ has been demonstrated to regulate pulmonary vascular function (Du et al., 2017; Yeligar et al., 2018), with loss of PPARγ resulting in increased PASMC proliferation (Du et al., 2017; Gien et al., 2014; Yeligar et al., 2018). The loss of PPARγ has also been shown to enhance mitochondrial‐derived H_2_O_2_ generation, decrease mitochondrial mass, and increase mitochondrial reactive oxygen species (ROS) generation (Klemm et al., 2011; Yeligar et al., 2018). In patients with pulmonary arterial hypertension, PPARγ expression has been found to be decreased in lung vascular lesions (Yeligar et al., 2018). Additionally, hypoxia has been shown to downregulate PPARγ expression in human PASMC in vitro and in mouse lungs in vivo, thereby stimulating PASMC proliferation and PH (Gien et al., 2014; Yeligar et al., 2018). Our experiments demonstrate that a loss of *Pde3a* from siRNA transfection led to a decrease in protein expression of PASMC PPARγ compared to controls (Figure 4a,g). Also, siPDE3B‐transfected WT PASMC significantly decreased PPARγ protein expression to similar levels as siPDE3A knockdown (Figure 4a,g). It is unclear whether the changes observed are due to direct effects from *Pde3b* knockdown or the associated decreases in PDE3A as a result of *Pde3b* knockdown. Similar to AMPK regulation, we postulate that the decrease in PDE3A following *Pde3b* knockdown may also account for the less robust changes in PPARγ protein expression compared with direct *Pde3a* knockdown. The regulation of this complex pathway by *Pde3* subtypes in the context of PH warrants further exploration.

We found that in PASMC, *Pde3a* deficiency by both siRNA transfection and PASMC isolated from knockout mice led to decreased phosphorylation of CREB with associated increased viable cell numbers (Figures 3c,d and 7). An important role for CREB activation in PASMC proliferation has been reported. Forced siRNA depletion of CREB was demonstrated to induce cell proliferation and hypertrophy (Klemm et al., 2011). Furthermore, mice with SMC‐specific CREB deletion were found to develop PH and CREB null SMC were shown to have increased cell proliferation (Garat et al., 2020). Interestingly, diminished activation of CREB was also observed in WT PASMC that were exposed to siPDE3A‐transfected adipocyte conditioned media, along with less robust but increased viable cell numbers (Figures 6b and 7), suggesting that elevated FFA, adipokines or inflammatory mediators due to elevated FFA may also be a contributing factor in PASMC proliferation.

We speculate that the elevation in FFA levels released by adipocytes deficient in *Pde3a* may be due to the activation of adipose tissue lipolysis via increased cAMP levels. Interestingly, although there was no difference in PDE3B protein expression in PASMC transfected with siPDE3A relative to scramble siRNA (Figure 2d), the combination of adipocyte media with siPDE3A‐transfected PASMC (Figure 5d,f) resulted in an increase in PDE3B protein expression. Free FA and/or secretory proteins within the adipocyte media appear to influence PDE3B protein expression; however, it is not clear which specific factors are regulating these changes. Exposure of scRNA‐transfected PASMC to siPDE3A‐transfected adipocyte conditioned media also resulted in an increase in PDE3B protein expression, corroborating the idea that FFA and/or secretory factors released into the adipocyte media can affect PASMC protein expression. Exposure to conditioned media from siPDE3A‐transfected adipocytes led to decreased AMPK and CREB activation in PASMC similar to those seen with direct PASMC *Pde3a* knockdown (Figure 6a,b). Likewise, α‐SMA, one of the contractile markers of differentiation and maturation of PASMC, was significantly lower in the scRNA‐transfected PASMC + siPDE3A‐transfected adipocyte conditioned media than those exposed to scRNA‐transfected adipocyte control media, albeit less so than with direct PASMC *Pde3a* knockdown (Figure 5e,f). This downregulation of α‐SMA suggests a switch to a dedifferentiated/pro‐proliferative phenotype (Lechartier et al., 2022). We postulate that the increased FFA, adipokines, secretomes, or lipotoxic metabolites released into the conditioned media may play a role in the observed alterations in the metabolic pathways (Ali Khan et al., 2018), but further studies will need to be performed to determine the precise mechanism involved. Nevertheless, the downstream effects of *Pde3a* deficiency on the release of mediators from other cell types, in this case adipocytes, prove to be an important factor in cell growth and viability.

Interestingly, cleaved caspase 3 levels were significantly elevated in *Pde3a*‐deficient PASMC (Figure 3e,f), *Pde3a*‐deficient PASMC exposed to adipocyte media (Figure 6c), and in scRNA‐transfected PASMC + siPDE3A‐transfected adipocyte conditioned media (Figure 6c). Although these findings may be indicative of increased apoptosis, the increased viable PASMC in our studies perhaps support other roles for caspase‐3, such as those of a nonapoptotic function including regulation of cell growth and homeostatic tissue maintenance (Eskandari & Eaves, 2022). Additional studies are necessary to determine the contributions of *Pde3a* deficiency to cell proliferation and apoptosis.

Future studies are needed to elucidate the specific role of PDE3A in metabolic pathways of not only PASMC, but also other cell types that may be controlled by paracrine regulation resulting in modulation of cellular energy metabolism and function that could contribute to PH pathobiology. The present study not only demonstrates subtype specific effects of the PDE3 subtypes on metabolic pathways and viability of PASMC, but also suggests a potential function of *Pde3* in maintaining homeostasis in PASMC.

## AUTHOR CONTRIBUTIONS

Dr. Paulina Krause contributed to design of the work, acquisition, and interpretation of data for the work, drafting and revising for intellectual content. Gabrielle McGeorge, Jennifer L. McPeek and Sidra Khalid contributed to design of the work, acquisition of the data, and drafting the work. Dr. Leif Nelin contributed to design of the work, analysis, and interpretation of data, and revising for intellectual content. Dr. Yusen Liu contributed to design of the work, analysis, and interpretation of data, and revising for intellectual content. Dr. Bernadette Chen contributed to conception of the work, analysis, and interpretation of data, and drafting and revising for intellectual content.

## FUNDING INFORMATION

This work was supported by grant 5 R01 HL136963 (BC) from the National Heart Lung and Blood Institute of the NIH.

## Supporting information


Figure S1.

## Data Availability

Data can be made available upon reasonable request to the corresponding author (BC). Full unedited Western blot images are openly available in figshare at https://doi.org/10.6084/m9.figshare.27170658.
