# Supplementary material for: Pde3a and Pde3b regulation of murine pulmonary artery smooth muscle cell growth and metabolism
Source: Physiol Rep. 2024 Oct 22;12(20):e70089. doi: 10.14814/phy2.70089 (PMC11494452; doi:10.14814/phy2.70089)

Supplement: Figure 1

Corresponds to Figure 2E:

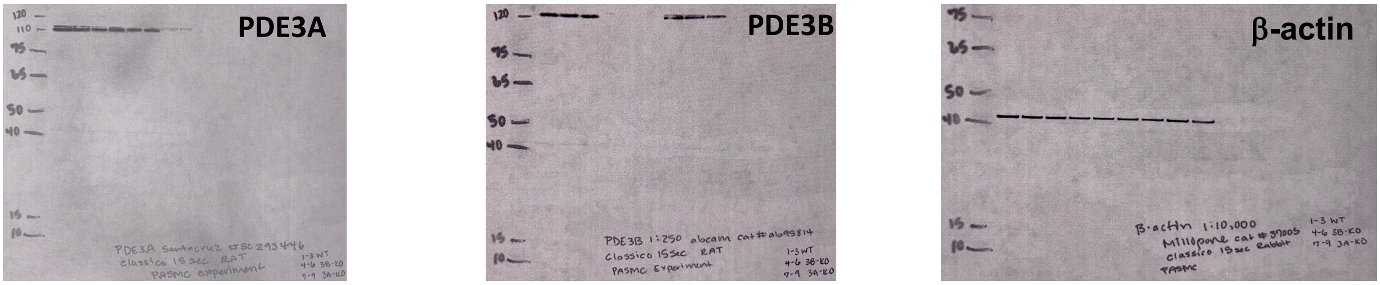

Corresponds to Figure 2F:

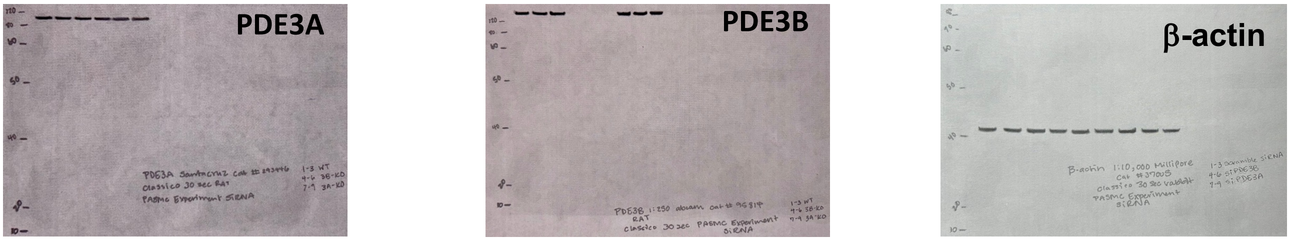

Corresponds to Figure 3A:

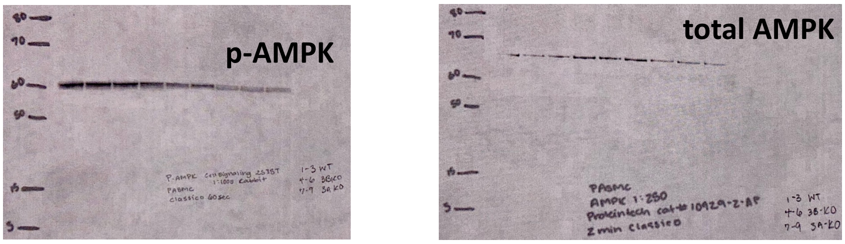

Corresponds to Figure 3B:

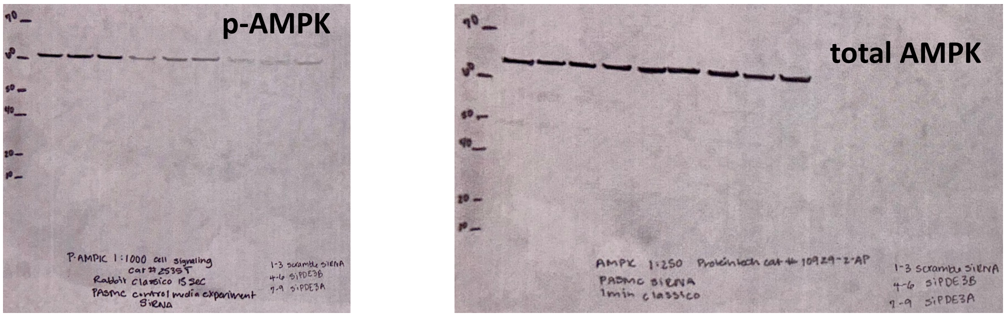

Corresponds to Figure 3C:

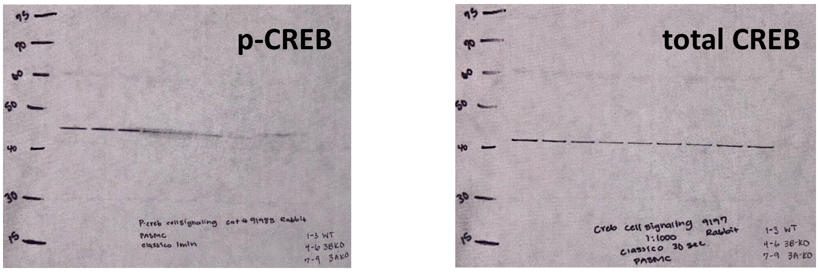

Supplement: Figure 2

Corresponds to Figure 3D:

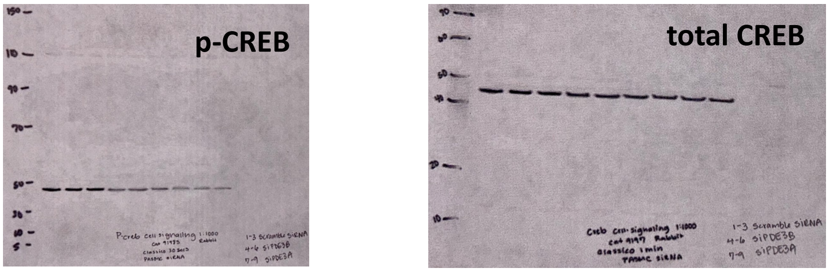

Corresponds to Figure 3E:

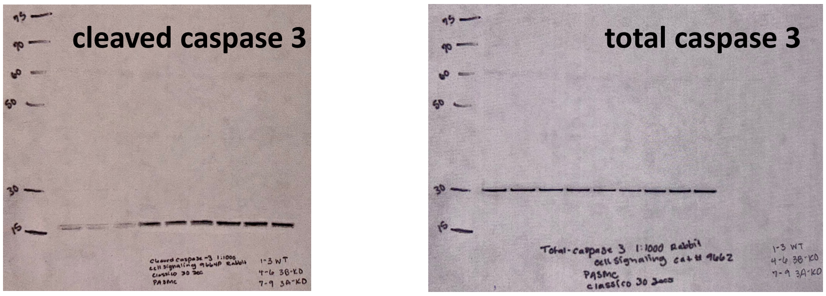

Corresponds to Figure 3F:

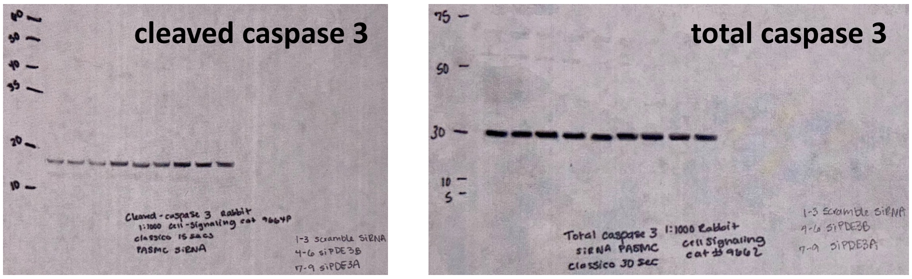

Corresponds to Figure 4G:

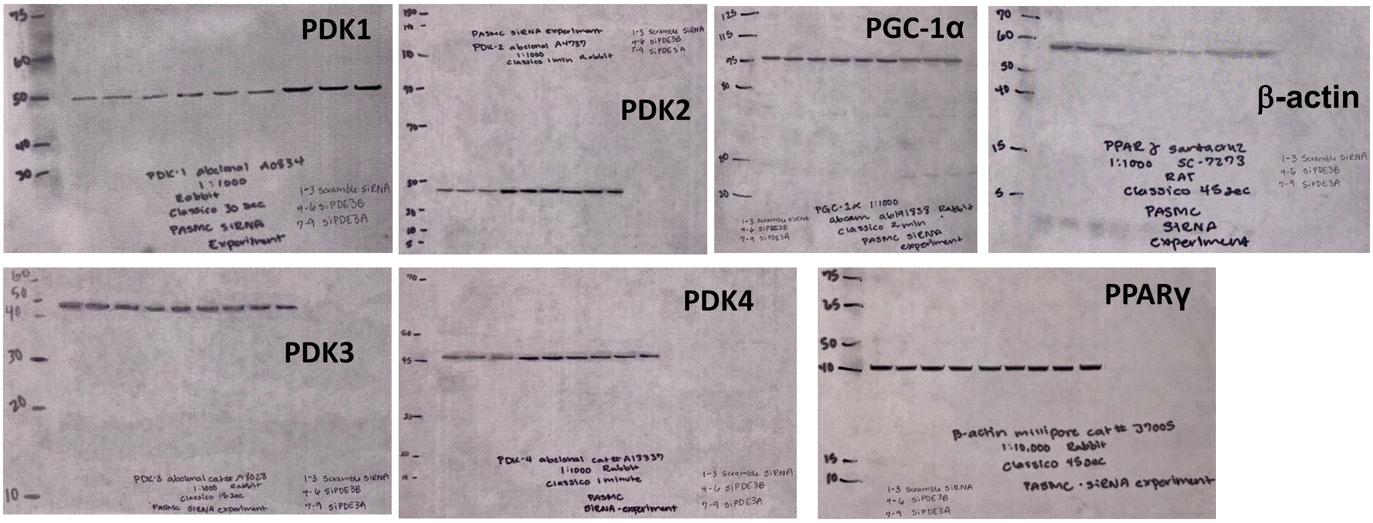

Supplement: Figure 3

Corresponds to Figure 5A:

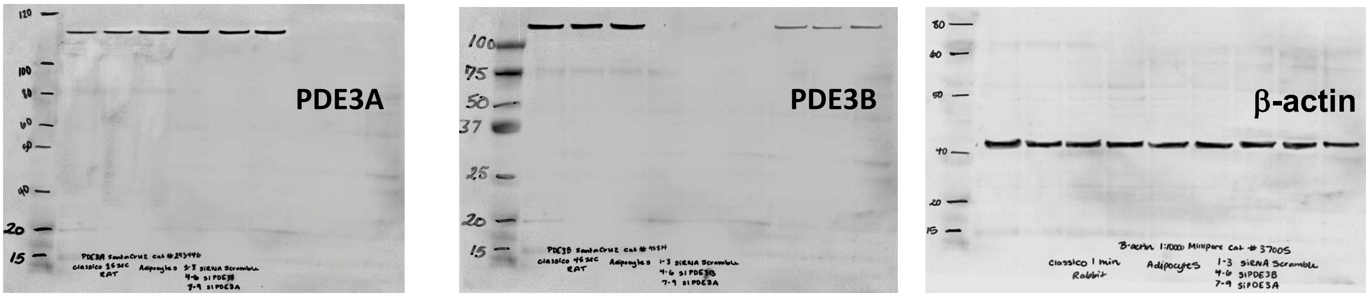

Corresponds to Figure 5F:

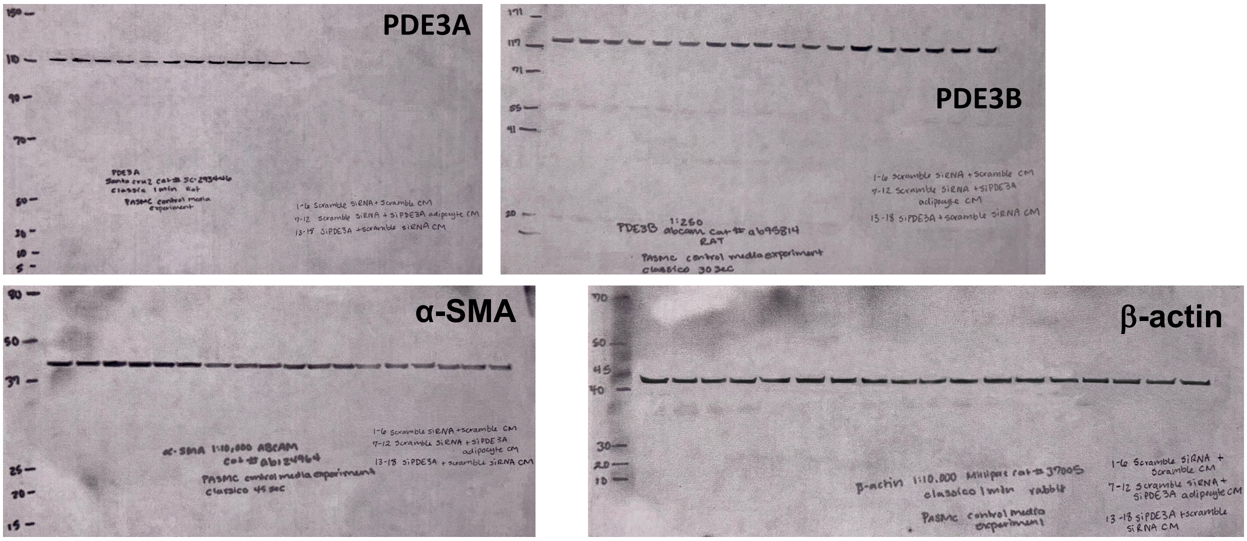

Supplement: Figure 4

Corresponds to Figure 6A:

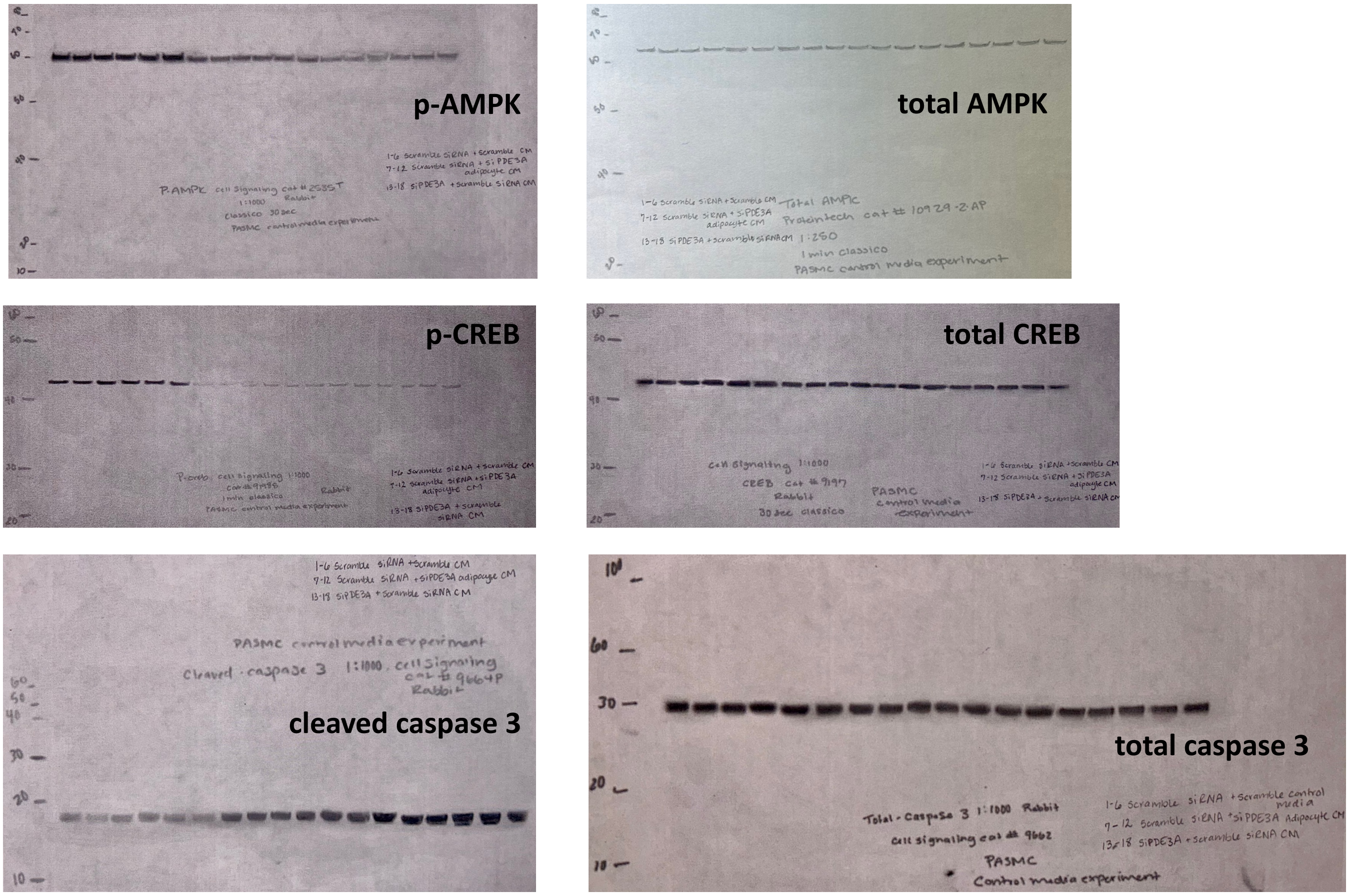

Supplement: Supplementary file 1 — Figure S1. [file PHY2-12-e70089-s001.pdf]
